# Adsorption Behavior of Polyelectrolyte onto Alumina and Application in Ciprofloxacin Removal

**DOI:** 10.3390/polym12071554

**Published:** 2020-07-14

**Authors:** Thi Huong Dao, Ngoc Trung Nguyen, Minh Ngoc Nguyen, Cao Long Ngo, Nhu Hai Luong, Duy Binh Le, Tien Duc Pham

**Affiliations:** 1 Faculty of Chemistry, University of Science, Vietnam National University, Hanoi, 19 Le Thanh Tong, Hoan Kiem, Hanoi 100000, Vietnam; daothihuong.k56@hus.edu.vn (T.H.D.); nguyenngoctrung_t61@hus.edu.vn (N.T.N.); nmngoc@hus.edu.vn (M.N.N.); 2 Department of Supplies and Warehouse, MPS, Vietnam, 80 Tran Quoc Hoan, Cau Giây, Hanoi 100000, Vietnam; nclong@iams.vast.vn; 3 Vietnam Academy of Science and Technology, 18 Hoang Quoc Viet, Cau Giay, Hanoi 100000, Vietnam; luonghai76@htd.vast.vn; 4 Institute of Propellant and Explosives, MOD, Vietnam, 192 Duc Giang, Long Bien, Hanoi 100000, Vienam

**Keywords:** PSS, alumina, adsorption isotherm, surface modification, ciprofloxacin

## Abstract

This study aims to investigate the adsorption behavior of a strong polyelectrolyte poly(styrenesulfonate) (PSS) onto alumina particles. Adsorption of PSS onto positively charged alumina surface increased with increasing ionic strength, indicating that non-electrostatic and electrostatic interaction controlled the adsorption. The removal of an emerging antibiotic ciprofloxacin (CFX) from water environment using PSS-modified alumina (PMA) was also studied. The removal of CFX using PMA was much higher than that using alumina particles without PSS modification in all pH ranges of 2–11. The removal of CFX reached 98% under the optimum conditions of pH 6, contact time of 120 min, adsorbent dosage of five milligrams per milliliter and ionic strength 10^4^-M NaCl. The adsorption isotherms of CFX at different salt concentrations fit well with a two-step adsorption model, while the adsorption kinetic fit well with a pseudo-second-order model with a good correlation coefficient (R^2^ > 0.9969). The CFX-removal from a hospital wastewater using PMA was more than 75%. Our study demonstrates that adsorption of PSS onto alumina to modify the particle surface is important to form a novel adsorbent PMA for CFX-removal from water environments.

## 1. Introduction

As versatile macromolecular materials, polymers are widely use in different applications such as biotechnology, drug delivery system, biosensor devices, cosmetics and environmental engineering, etc [1,2,3]. Polyelectrolytes are charged polymers that are often used for material modification in numerous applications including drug delivery [4] and wastewater treatment [5,6]. Therefore, polyelectrolyte adsorption onto solid surfaces is an important subject that is attracting the attention of many scientists.

Antibiotic resistance has become a serious problem across the globe in this century [7,8]. Antibiotic resistance is growing rapidly—especially in developing countries. One reason for the spread of antibiotic resistance is antibiotic residues in wastewater environment [9,10]. Therefore, a solution to reduce antibiotic resistance by removing antibiotics from aqueous solutions is of great importance. There are many methods have been investigated to treat antibiotics based on biologic, chemical, and physical processes or combined these techniques [11].

The enhancement of water quality is necessary in modem life which is based on applied science and technologies such as chemical [12], biologic [13], physical properties or combined together [14]. Many studies report the different techniques for antibiotic removal such as membrane processes, Fenton oxidation [15], photocatalytic degradation [16] and adsorption, etc. It has been found that adsorption is known as one of most effective methods for organic pollutants removal. In this, the modified adsorbent is an excellent candidate to obtain the high removal of charged molecules like antibiotics [17,18]. A novel adsorbent based on polyelectrolyte modified metal oxide particle for antibiotic removal is still a big challenge due the complicated adsorption behavior of antibiotic and adsorbent. The modification of metal oxide with an oppositely charged polyelectrolyte molecules can change the charging behavior of adsorbent and therefore enhance the removal of antibiotic.

To the best of our knowledge, this is the first study that investigates the adsorption of strong polyelectrolyte poly(styrenesulfonate) (PSS) onto alumina to fabricate a high performance adsorbent for removal of an antibiotic ciprofloxacin (CFX) from aqueous solution. The removal of CFX using PSS-modified alumina (PMA) at different conditions such as pH solution, adsorption time, adsorbent dosage and ionic strength is systematically studied. The application of PMA for CFX-removal from a real wastewater sample collected from hospital is also studied in this work.

## 2. Materials and Methods

### 2.1. Materials

High-purity (99.99%) α-Al_2_O_3_ particles with particle size of about 300 nm were purchased from AKP-30, Sumitomo, Japan. Poly(sodium 4–styrenesulfonate) (PSS)—with a molecular weight of 1000 kg/mol—was supplied by Sigma-Aldrich, Singapore. Ciprofloxacin (CFX) in the form of hydrochloride monohydrate (CAS 86393-32-0) > 98% (HPLC grade) was supplied from Tokyo Chemical Industry (Tokyo, Japan). Other chemicals— analytical-grade NaCl, HCl and NaOH— were delivered from Merck (Darmstadt, Germany). NaCl was used as ionic content in solution; HCl, NaOH were used to adjust the pH of the solutions. The alumina was treated following previous research [19]. Treated α-Al_2_O_3_ was added with different PSS-concentrations at pH 4. Then the suspensions were shaken for 2 h to modify the α-Al_2_O_3_ surface [19].

### 2.2. Adsorption Studies

The adsorption of PSS onto α-Al_2_O_3_ particles is detailed in our previous study [19]. The optimum conditions for PSS adsorption were adsorption time 120 min, mass ratio of PSS to alumina 20 mg/g and pH 4, using the batch-adsorption technique. The change in FT-IR results and the zeta potential confirmed the appearance of PSS onto α-Al_2_O_3_ [19]. In this research, the adsorption isotherms at different ionic strength were conducted and modeled by two-step adsorption model. The PSS-modified α-Al_2_O_3_ at optimum conditions is called PMA. The PSS solutions were separated using a refrigerated centrifuge (MR23i, JOUAN, France) with the speed of 12,000 rpm (5 °C) for 10 min. Concentrations of PSS were determined by molecular spectroscopy (UV-Vis). The adsorption capacity Γ (mg/g) of PSS or CFX onto α-Al_2_O_3_ or PMA was calculated by Equation (1):

(1) $$\Gamma =\frac{C_{i}-C_{f}}{m}$$

where *C_i_* (mg/L) is the initial concentration of PSS or CFX, *C_f_* (mg/L) are the final concentrations of PSS or CFX and *m* (mg/mL) is the adsorbent dosage.

For CFX adsorption, a stock solution of CFX was prepared by dissolved an accurate amount of CFX salt into 100 mL of ultrapure water to form a solution of 1000 mg/L. Each day, the working solution was diluted from the stock solution. The adsorption of CFX with α-Al_2_O_3_ or PMA was conducted by batch technique using an orbital shaker (OS-350D, Digisystem, Taipei, Taiwan) at room temperature 25 ± 2 °C, controlling by air-conditioned laboratory.

The removal (% R) of CFX was determined by Equation (2):

(2) $$\mathrm{Removal}\;\left(\%R\right)=\frac{C_{i}-C_{e}}{C_{i}}\times 100\%$$

where Ci and Ce are initial concentration and equilibrium concentration of CFX (mg/L), respectively. The experimental adsorption studies were carried out in triplicate and are shown with standard deviation error.

### 2.3. Analysis and Characterization

Spectrophotometry was used to determine concentration of PSS and CFX. The concentrations of PSS and CFX were calculated from linear relationship of absorbance and concentration of PSS and CFX by using a spectrophotometer (UV-1650 PC, Shimadzu, Kyoto, Japan) with a double of 10-mm path length quart cuvettes and the wavelength of 263 and 277 nm for PSS and CFX, respectively [20,21].

The surface functional groups of the materials were evaluated by Fourier-transform infrared spectroscopy FT-IR using an Affinity-1S spectrometer (Shimadzu, Japan). The change in functional groups in FT-IR spectra was used for confirmation of PSS onto α-Al_2_O_3._

### 2.4. Modeling by General Isotherm Equation

A general isothermal equation was used to fit adsorption isotherms of both PSS and CFX onto adsorbents. A two-step adsorption assumes that the adsorption follows two steps at the solid–liquid interface [22]. The equation is shown below:

(3) $$\Gamma =\frac{\Gamma_{\infty }k_{1}C\left(\frac{1}{n}+k_{2}C^{n-1}\right)}{1+k_{1}C\left(1+k_{2}C^{n-1}\right)}$$

where Γ is the adsorbed amount of CFX/PSS, C denotes the equilibrium concentrations of CFX/PSS in solution, k_1_ and k_2_ are equilibrium constants for the first layer adsorption and clusters of n molecules or multilayer adsorption and Γ_∞_ is the maximum adsorbed amount n.

The selected fitting parameters are described in our previously published papers [23,24,25].

### 2.5. Adsorption Kinetic

Pseudo-first-order and pseudo-second-order models were employed to evaluate the kinetic reaction order. The pseudo- first-order model is

(4) $$\log\left(q_{e}-q_{t}\right)=\log q_{e}-\frac{K_{1,k}}{2.303}t$$

where q_e_ is the maximum adsorption capacity, q_t_ is the equilibrium adsorption capacity at the time t, *K*_1,k_ is pseudo-first-order rate constant and t is adsorption contact time. The approximate minimum sum of square was applied for fitting model.

The pseudo-second-kinetic-order model was written as the equation:

(5) $$\frac{t}{q_{t}}=\frac{1}{K_{2,k}.q_{e}^{2}}+\frac{1}{q_{e}}t$$

where *K*_2,k_ is the pseudo-second-order rate constant. The pseudo-second-order model directly fit the experiment results with the interaction of valence force of solute and adsorbent.

## 3. Results and Discussion

### 3.1. Adsorption of PSS onto α-Al_2_O_3_

**Figure 1:**
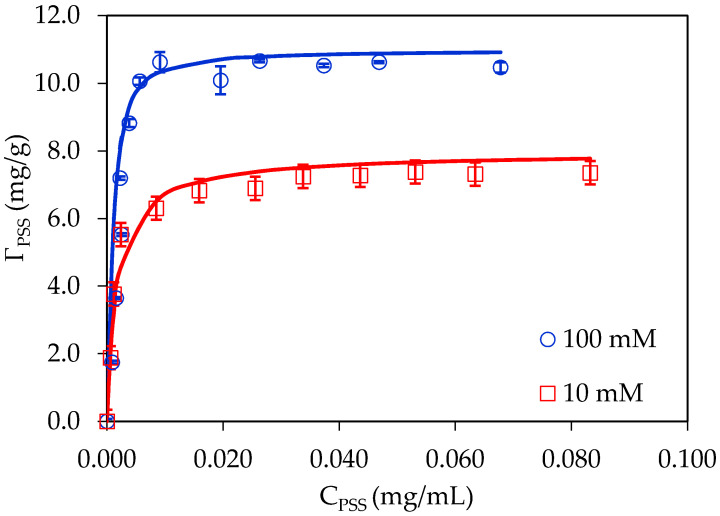
Adsorption isotherms of poly(styrenesulfonate) (PSS) onto α-Al_2_O_3_ at two NaCl concentrations (pH 4, adsorption time 120 min, mass ratio of PSS to alumina 20 mg/g). Points are experimental data while solid lines are the results of the two-step adsorption model. Error bars show standard deviations of three replicate.

The surface modification of PSS onto α-Al_2_O_3_ was studied in our previous publication [19]. In this research, the adsorption isotherms of PSS at two NaCl concentrations (pH 4) onto α-Al_2_O_3_ are shown in Figure 1. As can be seen in Figure 1, the PSS adsorption isotherms onto Al_2_O_3_ fit very well by the two-step adsorption model with the fit parameters shown in Table 1.

Figure 1 shows that adsorbed amount of PSS onto α-Al_2_O_3_ increased with increasing NaCl concentration from 10 to 100 mM. It is also observed that the adsorption amount of PSS onto Al_2_O_3_ at 100-mM NaCl was always higher than that at 10-mM NaCl. At high salt concentrations, the number of anions Cl^−^ (counter ions) increased, thus the electrostatic attraction between positively charged alumina surface and polyanion decreased [26]. Nevertheless, non-electrostatic interactions such as hydrophobic interaction, hydrogen bonding and Van der Waals forces—as well as the lateral interaction between PSS molecules may contribute to adsorption [27]. It should be noted that an increase of ionic strength may induce the *R*_h_ change of polyelectrolyte. For PSS, the *R*_h_ increased from 11.7 to 13.8 nm with an increase of ionic strength from 1 to 150 mM [28]. Polyelectrolyte adsorption at high ionic strengths may produce many counter ions to the bulk solution—thus the driving force for adsorption may be influenced by increasing entropy [29,30].

Table 1 and Figure 1 also indicate that at 10-mM NaCl, the maximum adsorbed amount of PSS $\left(\Gamma_{\infty }\right)$ for α-Al_2_O_3_ was 8.0 mg/g, while at 100-mM NaCl this value increased to 11.0 mg/g. This implies that, at high salt concentration the lateral and hydrophobic interactions may induce more loops and tails in the structure of adsorbed PSS onto Al_2_O_3_. As a result, the values of *k*_2,PSS_ were much higher at high salt compared with that at low salt. On one hand, the values of *k*_1_ (denoting for first layer adsorption) did not significantly change. Therefore, the PSS-adsorbed layer was less flat at higher salt concentrations than that at low salt concentrations. As the resulted, PSS adsorption capacity increased with an increase of salt concentration. The results here are similar to polyelectrolyte adsorption onto cotton fiber, in which adsorption with a high value of *k*_2_ with a multilayer formation were found [31].

**Figure 2:**
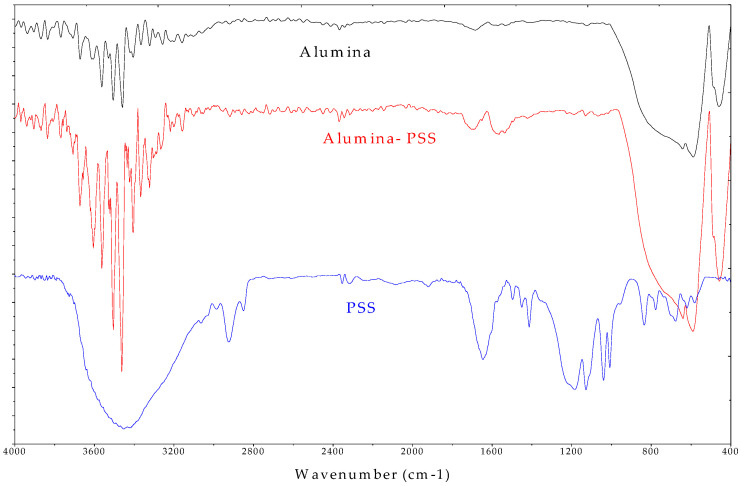
FT-IR spectra of alumina and alumina modified with PSS (alumina–PSS) and PSS.

Figure 2 shows the FTIR spectra of Al_2_O_3_ before and after PSS adsorption The asymmetric and symmetric bands of PSS at 1185 and 1039 cm^−1^ [32], did not appear at the FT-IR of Al_2_O_3_ while a small symmetric band of 1039 cm^−1^ shifted to longer wavenumber of 1130 cm^−1^ on the surface of Al_2_O_3_ after PSS adsorption. Furthermore, the stretching of C–S groups in PSS molecules at 676 cm^−1^ and the boarding peaks at about 3550 cm^−1^ assigned for O–H [33], changed to the various peaks at the wavenumber of 3400–3600 cm^−1^ in FT-IR spectra of Al_2_O_3_ after PSS adsorption. This suggests that hydrogen bonding, lateral interaction and electrostatic attraction may contribute to the PSS adsorption onto α-Al_2_O_3_.

The results of adsorption isotherm and FT-IR spectra indicate that the PSS adsorption onto α-Al_2_O_3_ surface achieved the maximum adsorption amount of 11 mg/g at 100-mM NaCl, contact time 120 min and pH 4, as shown in our previous publication [19]. The PMA was formed with the initial PSS concentration of 0.1 mg/mL.

### 3.2. Optimization of Effective Parameters for CFX-Removal

#### 3.2.1. Effect of pH on CFX-Removal Using α-Al_2_O_3_ and PSS-Modified α-Al_2_O_3_ (PMA)

**Figure 3:**
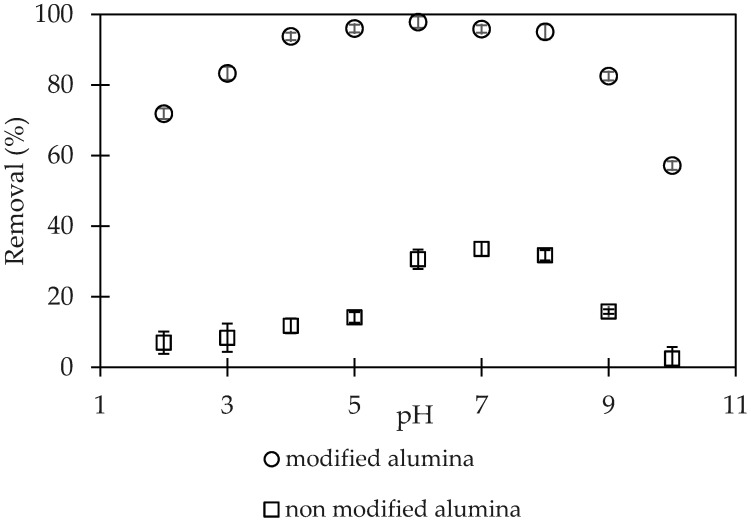
Effect of pH on ciprofloxacin (CFX) removal using α-Al_2_O_3_ (non-modified alumina) and PMA (modified Al_2_O_3_) from pH 2 to 10. (Ci (CFX) = 10 mg/L, contact time 90 min, adsorbent dosage 5 mg/mL, 1-mM NaCl). Error bars show standard deviation of three replicates.

The effect of pH on CFX-removal using α-Al_2_O_3_ and PSS-modified α-Al_2_O_3_ (PMA) is shown in Figure 3. As can be seen, the highest removed CFX efficiency was achieved at pH 6, which was about 98% when using PMA. On one hand, at low pH, the α-Al_2_O_3_ may have been dissolved, so that the removal of CFX decreased. On the other hand, under basic conditions, the CFX formed the negative species that induced the repulsive electrical force between adsorbent and CFX. Furthermore, the desorption of PSS out to PMA may occur at high pH. Figure 3 shows the pH optimum for CFX-removal is 6.0 which is closed to *pK*_a1_ of CFX [34]. Figure 3 also indicate that CFX-removal using α-Al_2_O_3_ was extremely low efficiency compared with PMA at all pH range. The highest CFX-removal at pH 7 was only 40%, while the maximum CFX using PMA removal reached to 98%. This implies that PMA was much better adsorbent than raw α-Al_2_O_3_ for CFX-removal.

#### 3.2.2. Effect of Ionic Strength on CFX-Removal Using α-Al_2_O_3_ and PMA

Ionic strength can influence to electrostatic interactions [22]. CFX-removal using α-Al_2_O_3_ and PMA was conducted at pH 6, adsorbent dosage 5 mg/mL and contact time 90 min—with different ionic strength from 0- to 100-mM NaCl.

**Figure 4:**
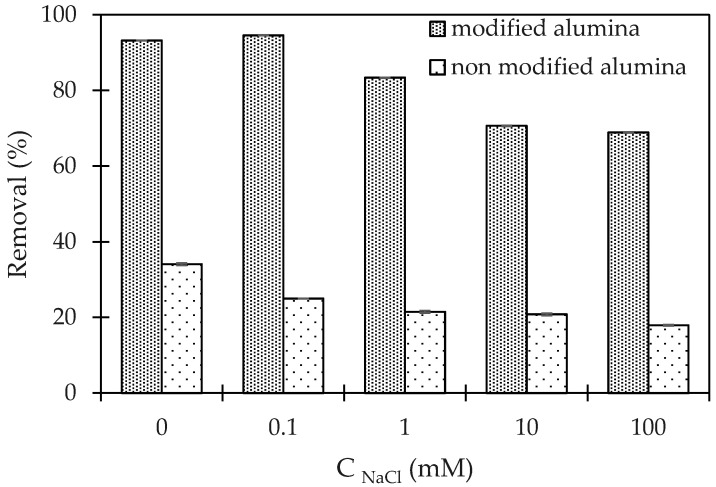
Effect of ionic-strength CFX-removal using α-Al2O3 (non-modified alumina) and PMA (modified alumina). (Ci (CFX) = 10 mg/L, contact time 90 min, adsorbent dosage 5 mg/mL, pH 6). Error bars show standard deviation of three replicates.

Figure 4 shows that an increase of NaCl concentration induced a decrease of CFX-removal. At high salt concentrations, the electrostatic attraction was screened while the removal of CFX decreased due to the decrease of electrostatic interaction. The effect of ionic strength for the cases of non-modified alumina and modified alumina showed similar trends. At different ionic strengths, CFX-removal using PMA was much higher than that using non-modified alumina. Therefore, further studies focus only on the PMA, which was better than α-Al_2_O_3_ without PSS.

#### 3.2.3. Effect of Contact Time on CFX-Removal Using PMA

**Figure 5:**
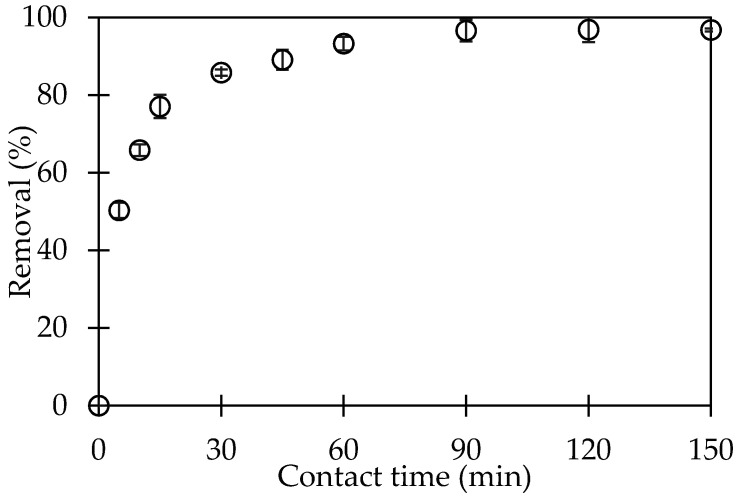
Effect of contact time on CFX-removal by using PSS-modified alumina (PMA). (Ci (CFX) = 10 mg/L, adsorbent dosage 5 mg/mL, pH 6, 1-mM NaCl). Error bars show standard deviation of three replicates.

The time to achieve the equilibrium for CFX adsorption process is known as the contact time. The effect of contact time for CFX-removal with the different time intervals is shown in Figure 5.

As shown in Figure 5, CFX-removal increased with an increase of contact time from 0 to 90 min. After 90 min shaking, CFX-adsorption onto PMA reached an equilibrium with about 97% of CFX-removal. Thus, the CFX-adsorption onto PMA could reach saturation after 90 min. After this, CFX-removal changed insignificantly. The contact time of 90 min was much shorter than other materials in previously published papers [34,35]. Therefore, the optimum contact time for CFX-removal using PMA is 90 min that was kept for the next studies on CFX-removal using PMA.

#### 3.2.4. Effect of Adsorption Dosage on CFX-Removal Using PMA

**Figure 6:**
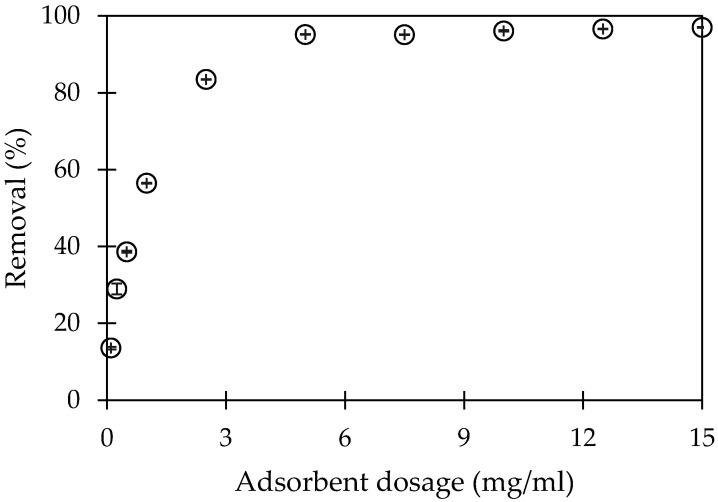
Effect of adsorbent dosage on CFX-removal by using PMA. (Ci (CFX) = 10 mg/L, contact time 90 min, pH 6, 1-mM NaCl). Error bars show standard deviation of three replicates.

The adsorbent dosage is important parameter which strongly influences to the total surface area and surface-charge density. The result of ionic strength effect suggests that the main interaction in CFX-adsorption onto PMA is electrostatic attraction, which is depended on charge density. Various adsorbent dosages of PMA ranging from 0.1 to 15-mg/mL were used to remove CFX. The results are indicated in Figure 6.

Figure 6 shows that CFX-removal increased significantly with the 0.1 to 5-mg/mL adsorbent dosage. Then, CFX-removal remained stable with increasing adsorbent dosage. The total surface area and charge density increased with increasing adsorption dosage. However, CFX-removal did not increase for a larger amount of adsorbent dosage of greater than 5 mg/mL due to the fact that CFX-adsorption amount onto the PMA surface was saturated. Therefore, the optimum adsorbent dosage for CFX-removal using PMA was 5 mg/mL.

### 3.3. Adsorption Isotherms of CFX onto PMA

**Figure 7:**
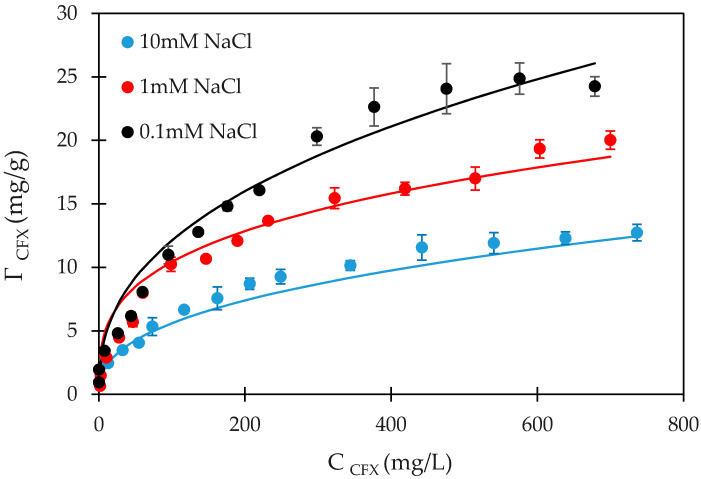
Adsorption isotherms of CFX onto PMA at different NaCl concentrations. Points are experimental results while solid lines are fitted by the two-step adsorption model. Error bars show standard deviation of three replicates.

The effect of ionic strength onto CFX-adsorption with various initial concentrations was clearly observed on the isotherms (Figure 7). Adsorption isotherms show the effects of ionic strength and initial concentration. Figure 7 shows the adsorption capacity as the function of CFX concentrations from 5 to 800 mg/L at 0.1-, 1- and 10-mM NaCl under the optimum conditions of pH 6, contact time 90 min and adsorbent dosage of 5 mg/mL.

As discussed in the section on the effect of ionic strength, the CFX-removal decreased with increasing NaCl concentration with a low CFX concentration of 10 mg/L. Herein, the adsorption isotherms with different initial CFX concentrations achieved the similar trend. The adsorption of CFX onto PMA reduced with an increase of ionic strength. The high ionic strength caused the increase in the number of positive cations (counter ions) and decrease of the electrostatic attraction between cationic species CFX and negatively charged PMA surface. The counter ions Na^+^ in the solution caused the electrical double layer decreased so that the net charge of PMA was decreased [36]. Other interactions such as hydrophobic interaction, Van der Waals and hydrogen bonding known as non-electrostatic ones could induce the adsorption at high salt concentration. However, the adsorption capacity of CFX at high salt was much smaller than that at low salt, representing that the adsorption isotherm at low salt was always above the isotherm at high salt. This indicates that CFX-adsorption onto PMA is induced by electrostatic attraction rather than non-electrostatic interactions.

Figure 7 also indicates that the experimental data fit well with the two-step adsorption model using the fit parameters shown in Table 2. The CFX-adsorption isotherms onto PMA was fitted well by a two-step model. The values of k1 were obtained from adsorption at low concentrations of CFX by applying the Langmuir equation. The variables of *k*_2_ and n were determined by trials and error method. At the high salt concentration (10 mM), the *k*_1,CFX_ value which was found to be 2 × 10^4^ (g/mg^−1^) was 11 times greater than that at low salt concentration (0.1 mM). It implies that the active sites for adsorption of CFX increased with decreasing salt concentration. In other words, the higher value of *k*_1,CFX_ obtained, the stronger was the electrostatic attraction induced CFX-adsorption. The maximum adsorption capacity at 0.1 mM was 2 times higher than that at 10-mM NaCl. The *n*_CFX_ was the same while the value of *k*_2,CFX_ changed insignificantly for all cases. The error bars shown the standard deviations of different replicates were reasonable and close to solid line, indicating that the model was suitable to represent adsorption isotherm of CFX onto PMA. Based on the fitting parameters, it can be found that the CFX-adsorption occurred onto PMA surface by a monolayer than the multilayer formation that is similar trends in previous publications [36,37,38].

### 3.4. Adsorption Kinetic of CFX onto PMA

The CFX-kinetic adsorption onto PMA was studied with three different CFX initial concentrations of 10, 50, 250 mg/L, under the optimum adsorption conditions.

**Figure 8:**
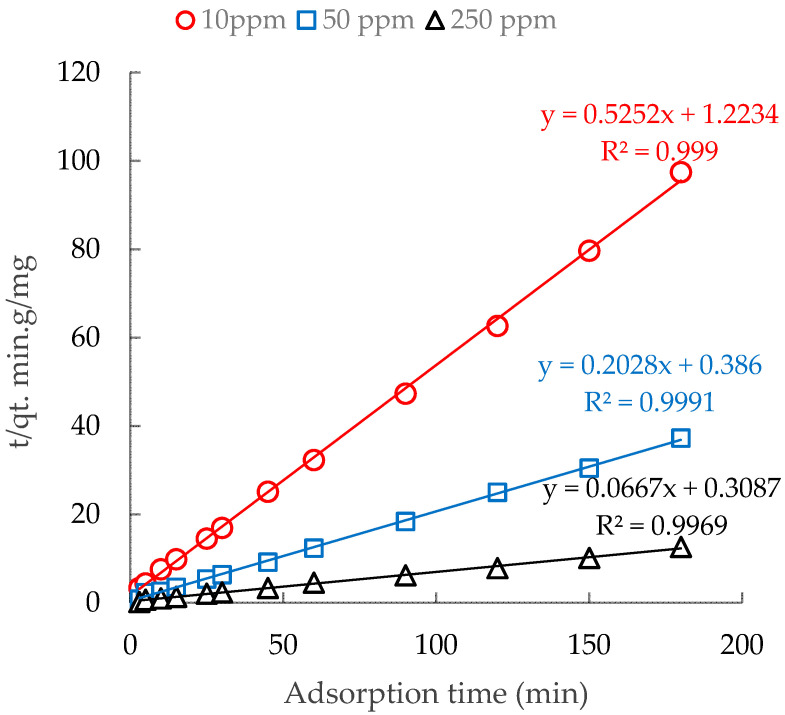
Pseudo-second-order model for CFX-adsorption kinetic onto PMA with three initial CFX concentrations.

Table 3 shows the rate constant (*K*), adsorption capacity (*q*_e_) and correlation coefficients (*R*^2^) of the pseudo-first-order and pseudo-second-order models. The effectiveness of model was evaluated through the *R*^2^ values. As can be seen, the pseudo-second-order model fit the adsorption kinetic of CFX better than the pseudo-first-order one, indicating that the adsorption kinetic of CFX onto PMA is in accordance with pseudo-second-order. The linear fits based on the experimental data are shown in Figure 8.

Figure 8 shows that adsorption kinetics of CFX onto PMA at three initial CFX concentrations fitted by pseudo-second-order achieved high R^2^ values (>0.9969). The value of K_2_ decreased from 0.225 to 0.014 when the initial CFX concentrations increased from 10 to 250 mg/L. The decrease in the adsorption rate constant represents the adsorption kinetics for higher initial concentration of CFX because of limited number of adsorption sites of PMA. Our results here are similar to adsorption of organic molecules onto surface modified adsorbents [17,19,25,36].

### 3.5. Regeneration Study

The regeneration of adsorbent is a key role to evaluate the stability and reusable property. The various type of desorption solutions including 0.1-M NaOH, 0.1-M HCl and methanol (MeOH) were used to conduct the desorption process. Almost CFX could desorb using NaOH one time then it is decreased rapidly with the next desorption. The desorption when using HCl was about 50% then it decreased slightly with the more desorption time. On the other hand, CFX could not desorb when using MeOH (not shown in detail).

**Figure 9:**
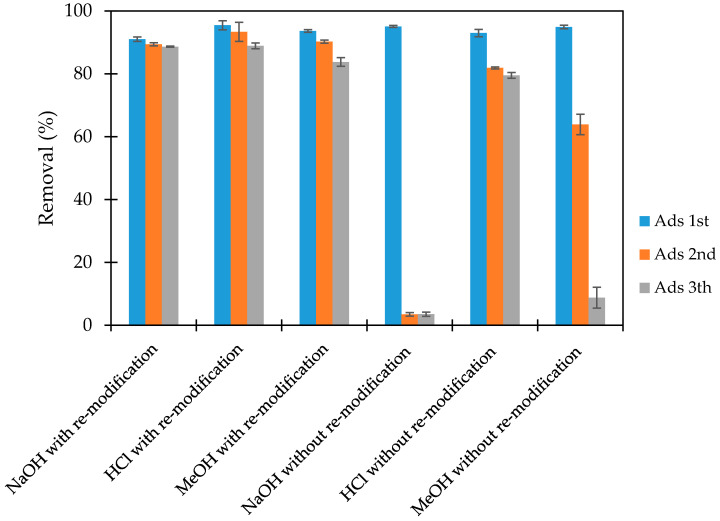
Regeneration of adsorbent with remodification and without remodification using different solutions NaOH, HCl, MeOH. Error bars show standard deviation of three replicates.

PMA after desorption was used to conduct CFX-removal again to evaluate the generation potential of the adsorbent (Figure 9). Figure 9 shows that the CFX-removal after regeneration still reached greater than 80%, and it changed insignificantly after three times of reuse. For NaOH, the CFX-removal slightly decreased after reuse of the adsorbent. The results show that PSS desorption could be occurred simultaneously with CFX desorption. When using MeOH, CFX-removal decreased too much at the third time of CFX-removal. The MeOH solution cannot be used for CFX desorption that may be explained by the unsaturated adsorption of modified PMA. Because CFX-adsorption occurs on the positively charged PMA surface, the highest desorption will take place with a basic solution. On one hand, HCl can produce negatively charged ions (Cl^−^), so that a competition of adsorption occurs that can promote the CFX-adsorption. On the other hand, the interaction of MeOH with CFX on the adsorbent is hydrophobic interaction that is not strong enough to desorb the CFX from the adsorbent. The generation study indicate that the PMA is reusable adsorbent for CFX-adsorption.

### 3.6. Application for CFX-Removal from Hospital Wastewater

The optimum conditions for CFX-removal were applied for the treatment of an actual wastewater sample in this part. There are many complicated pollutants in real wastewater that strongly influences to performance of adsorbent. Therefore, the experimental evaluation of real sample treatment is important. Actual samples of wastewater were taken from a hospital in Hanoi. The wastewater samples were then kept stored at low temperature in a cooling refrigerator within 7 days of the experiment. Samples were pretreated by filtering to remove the tiny particles. As seen by the results, the optimum conditions for CFX-removal were obtained when the SMN dosage was kept at 5 mg/mL and the pH of wastewater was 7.0 (checked after pretreatment) and contact time 90 min for real sample a treatment application.

**Figure 10:**
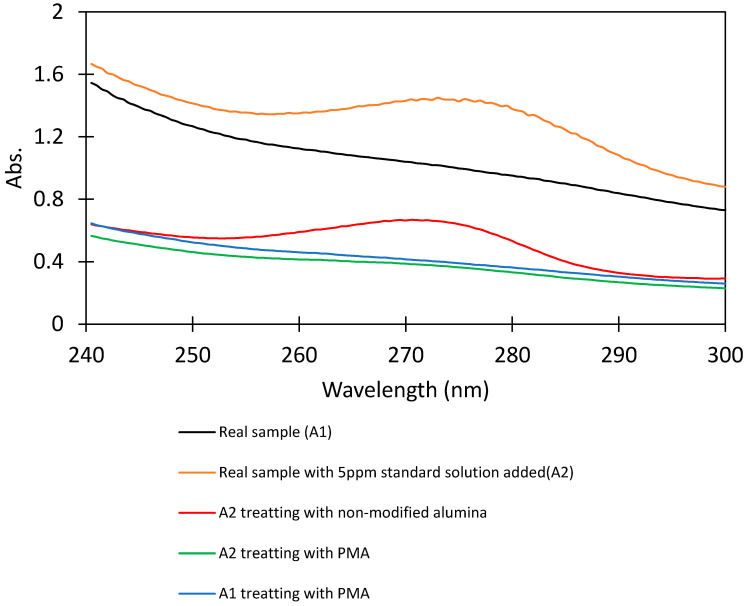
UV-Vis spectra of CFX in the hospital wastewater samples before and after treatment. The (A1) real sample and the (A2) real samples with 5 ppm of CFX standard solution added were treated using alumina and PMA. Baseline subtraction was conducted at the wavelength of CFX maximum absorbance, 272 nm.

In this section, we try to remove CFX from the actual hospital wastewater. Figure 10 shows that the high background containing many contaminants in the UV spectra (A1) was significantly reduced after treatment with PMA, indicating that PMA can remove not only CFX, but also many contaminants that appear in wastewater. This trend is similar for the case of the addition of 5 mg/L of CFX into a real sample (A2). By calculations, the CFX-removal efficiency achieved greater than 75% when using PMA while only 30% of pollutant molecular was removed by using alumina without surface modification. Our results again indicate that PMA is a novel adsorbent and high performance for antibiotic removal from wastewater solution.

## 4. Conclusions

This paper reports a new study of PSS adsorption onto alumina particles and application in CFX-removal from water environment. The adsorption of PSS increased with increasing ionic strength due to the both electrostatic and non-electrostatic interactions and the adsorption capacity reached to 11.0 mg/g. The removal of CFX achieved greater than 98% while the maximum adsorption capacity of 28.02 mg/g was achieved under the optimum conditions of pH 6, contact time 90 and adsorption dosage of five milligrams per milliliter. The maximum CFX-adsorption capacity was found to be 25 mg/g. The PMA was applicable for CFX-removal from an actual hospital wastewater with the removal efficiency of 75%. Our results indicate that PSS adsorption plays a key role to form a novel adsorbent PMA for antibiotic removal from aqueous solution.

## Figures and Tables

**Table 1:** Fit parameters for the adsorption isotherms onto Al_2_O_3_ at different NaCl concentration (pH 4). The maximum adsorbed amount $\Gamma_{\infty ,\mathrm{PSS}}\;$, the equilibrium constants *k*_1,PSS_, *k*_2,PSS_ for first layer and multilayer, respectively, *n* the number of clusters of PSS molecules.

C _NaCl_ (mM)	$\Gamma_{\infty ,\;\mathrm{PSS}}\;(\mathrm{mg}/g)$	*k*_1,PSS_ (g/mg)	*k*_2,PSS_ (g/mg)^n−1^	*n*_PSS_
100	11.0	900	1000	1.9
10	8.0	1000	150	1.9

**Table 2:** Fit parameters for the adsorption isotherms of CFX onto PSS-modified Al_2_O_3_ (PMA) at different NaCl concentrations (pH 6). The maximum adsorbed amount is $\Gamma_{\infty ,\;\mathrm{PSS}}\;$, the equilibrium constants are *k*_1,CFX_, *k*_2,CFX_ for first layer and multilayer, respectively, *n* the number of clusters of CFX molecules.

C _NaCl_ (mM)	$\;\Gamma_{\infty ,\;\mathrm{PSS}}\;(\mathrm{mg}/g)$	*k*_1,CFX_ (10^4^ g/mg)	*k*_2,CFX_ (g/mg)^n−1^	*n*_CFX_
0.1	28.02	22	1260	1.4
1	19.92	9	1249	1.4
10	13.02	2	1250	1.4

**Table 3:** Parameters of adsorption kinetics of ciprofloxacin (CFX) onto PMA.

*C*i (mg/L)	Pseudo-First-Order			Pseudo-Second-Order		
	*K*_1,k_ (1/min)	*q*_e_ (mg/g)	*R* ^2^	*K*_2,k_ (g/mg.min)	*q*_e_ (mg/g)	*R* ^2^
10	0.146	1.835	0.9863	0.225	1.904	0.9990
50	0.131	4.879	0.9984	0.106	4.930	0.9991
250	0.124	13.865	0.9686	0.014	14.992	0.9969
